# Supplementary material for: Bergamottin and PAP-1 Induced ACE2 Degradation to Alleviate Infection of SARS-CoV-2
Source: Int J Mol Sci. 2022 Oct 19;23(20):12565. doi: 10.3390/ijms232012565 (PMC9604380; doi:10.3390/ijms232012565)
Supplement: Supplementary file 1 [file ijms-23-12565-s001.zip › ijms-1927731-supplementary.pdf]

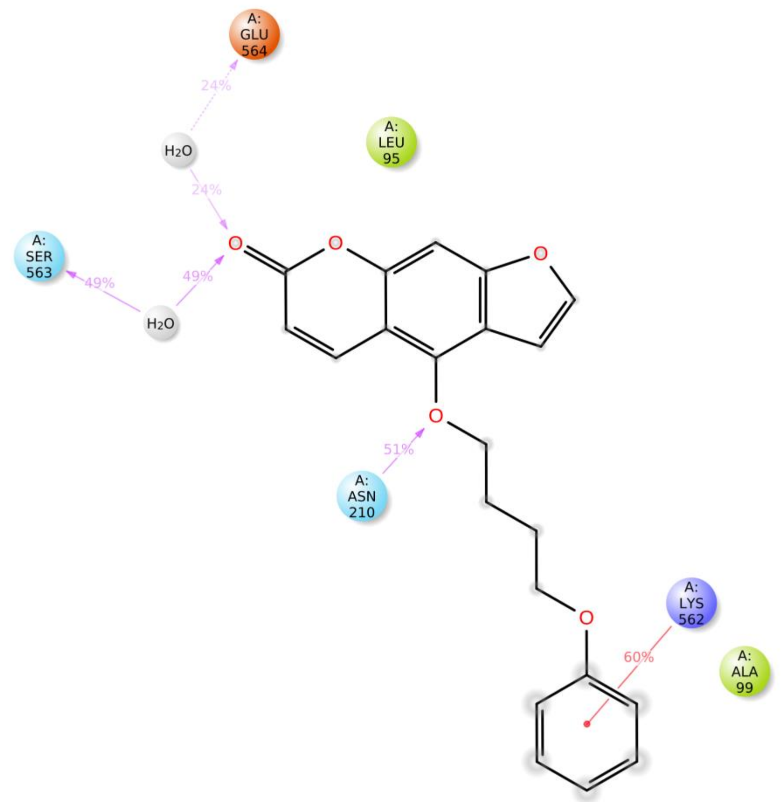

Supplement Figure S1 Interaction Analysis of PAP-1 and ACE2.

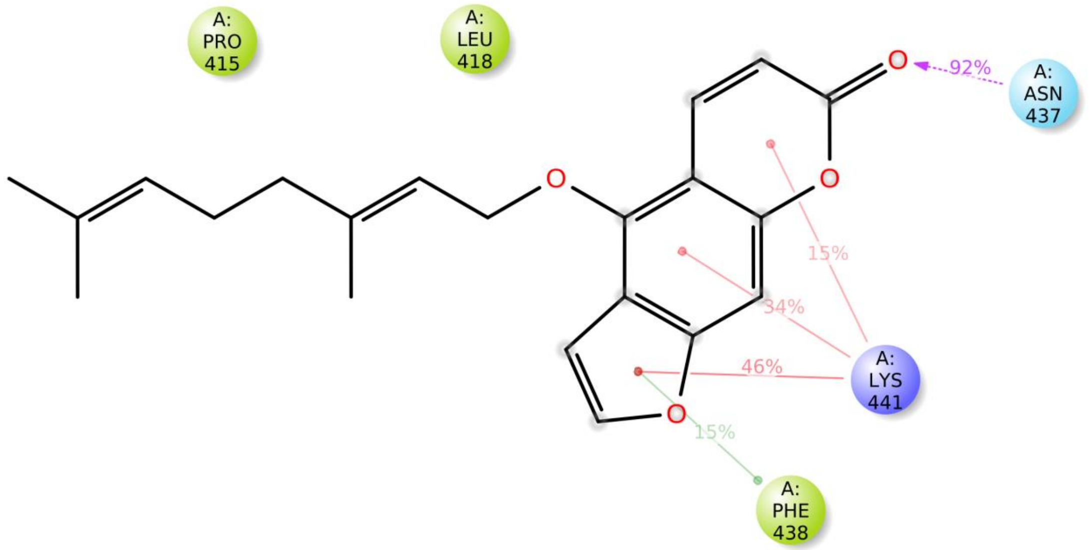

Supplement Figure S2 Interaction Analysis of Bergamot and ACE2.
